# Comparative analysis between high*-*grade serous ovarian cancer and healthy ovarian tissues using single-cell RNA sequencing

**DOI:** 10.3389/fonc.2023.1148628

**Published:** 2023-04-14

**Authors:** Xiao Zhang, Shihao Hong, Chengying Yu, Xiaozhong Shen, Fangying Sun, Jianhua Yang

**Affiliations:** ^1^ Department of Obstetrics and Gynecology, Sir Run Run Shaw Hospital, School of Medicine, Zhejiang University, Key Laboratory of Reproductive Dysfunction Management of Zhejiang Province, Hangzhou, China; ^2^ Department of Obstetrics and Gynecology, Longyou People’s Hospital, Quzhou, China; ^3^ Medical School, Nantong University, Nantong, China

**Keywords:** single-cell RNA-seq, ovarian cancer, human cancer, transcriptomics, differential analysis

## Abstract

**Introduction:**

High-grade serous ovarian cancer (HGSOC) is the most common histological subtype of ovarian cancer, and is associated with high mortality rates.

**Methods:**

In this study, we analyzed specific cell subpopulations and compared different gene functions between healthy ovarian and ovarian cancer cells using single-cell RNA sequencing (ScRNA-seq). We delved deeper into the differences between healthy ovarian and ovarian cancer cells at different levels, and performed specific analysis on endothelial cells.

**Results:**

We obtained scRNA-seq data of 6867 and 17056 cells from healthy ovarian samples and ovarian cancer samples, respectively. The transcriptional profiles of the groups differed at various stages of ovarian cell development. A detailed comparison of the cell cycle, and cell communication of different groups, revealed significant differences between healthy ovarian and ovarian cancer cells. We also found that apoptosis-related genes, URI1, PAK2, PARP1, CLU and TIMP3, were highly expressed, while immune-related genes, UBB, RPL11, CAV1, NUPR1 and Hsp90ab1, were lowly expressed in ovarian cancer cells. The results of the ScRNA-seq were verified using qPCR.

**Discussion:**

Our findings revealed differences in function, gene expression and cell interaction patterns between ovarian cancer and healthy ovarian cell populations. These findings provide key insights on further research into the treatment of ovarian cancer.

## Introduction

Ovarian cancer is one of the most common gynecologic malignancies in the world, with dismal prognosis (1). High-grade serous ovarian cancer (HGSOC) is the most aggressive type of ovarian cancer (2). High-grade ovarian serous cancer is associated with poor survival rates compared with early-stage and high-grade cancers, with the 5-year survival rate being only 27% (3). Advanced high-grade serous ovarian cancers tend to invade adjacent organs, metastasizing to the peritoneum and lymph nodes (4). So far, studies of high-grade serous ovarian cancer and the discovery of long-term effective treatment strategies for this disease are limited. Therefore, there is need for in depth research into the regulation mechanisms of genes associated with progression of high-grade ovarian cancer. Data from high throughput sequencing technologies indicate that many human genes are transcribed into RNAs, but only a small part of RNAs is finally translated into proteins (5, 6). Genome information flows through various molecular layers, including epigenome, transcriptome, proteome, and metabolome, to produce characteristic traits (7). As a result, we have gained a deeper understanding of the molecular complexity of ovarian cancer, especially the complexity of the genome. RNA-seq is a technique used to analyze RNA expression in whole tissues. However, this approach does not highlight contributions from different cell types (8). Single-cell RNA sequencing (scRNA-Seq) technologies provide essential opportunities to study cellular heterogeneity on the gene level (9).

Single-cell sequencing technology involves separation of groups of cells within tissues and body fluid into single cells, and analyzing their genetic materials using high-throughput sequencing techniques to reveal cellular heterogeneity among different tissues and cell types (10, 11). Each single cell found within high‐grade serous ovarian cancer has unique microenvironment, transcriptomic and epigenomic characteristics (12). Although cells contain the same genes, differences in mechanisms of transcriptional modulation drives stochastic gene expression. RNA sequencing (RNA-seq) is a bulk sequencing technique that analyzes the molecular complexity of tumor environment based on the average expression level of different cells, and cannot reveal the internal differences between different cell subsets (13). Single-cell sequencing differs from conventional tissue sequencing because it involves genome or transcriptome sequencing of nucleic acid (DNA or RNA) in a single cell, which is useful for identifying new markers, rare subpopulations and evolutionary patterns (14). ScRNA-seq can be used to determine the effect of gene expression on genetic structure diversity (15), individual cell level and interaction with host immune system in tumors (16). The analysis of single cell transcriptome RNA in a single tumor sample is especially important for understanding the cells in the cancer microenvironment. ScRNA-seq has become an indispensable part of the scientific research process. It can dissect tumor tissues into various cell types or cell shapes, and characterize tumor tissues (17). Clinically, it provides new insights into pharmacological mechanisms and provides new targets for tumor treatment.

In this study, we aimed to identify the potential key genes and pathways associated with HGSOC progression using single-cell transcriptome-specific analysis. We first determined the specific proportions of cell populations and special subpopulation of endothelial cells utilizing the data in the GEO public database. Next, we systematically analyzed signaling pathways involved in cellular function in HGSOC. Meanwhile, we characterized different cell interaction patterns in HGSOC and normal ovarian tissues through Cellchat analysis. We further identified differentially expressed genes *via* Gene ontology (GO) analyses, then we verified the reliability of the ten most differential expression of mRNAs by quantitative real-time PCR (qPCR) in clinical samples of HGSOC and in normal tissues. This research could help to understand tumor heterogeneity at the transcriptome level and the mechanisms of ovarian cancer metastasis and refractory to treatment may have major implications for therapeutic development and patient survival.

## Materials and methods

### Data sources and collection of human samples

We collected tumor and normal samples from ovarian cancer patients at Zhejiang University Sir Run Run Shaw Hospital, all detailed information of patients are listed in **Supplementary Table 1**. We downloaded two datasets, GSE147082 (18), and GSE118127 (19), which consisted of scRNA-seq data from Gene Expression Omnibus (GEO; https://www.ncbi.nlm.nih.gov/geo/) database.

### Data integration and analysis

The basic analysis steps of single-cell transcriptome were based on the R package Seurat (https://satijalab.org/seurat/, v.3.2.0) (20, 21). We read in the relevant single cell transcriptome matrix through the Read10X function, and set the following quality control standards: 1000<nFeature_RNA<6000, percent.mt<10. We normalized the data by LogNormalize method to eliminate the influence of library size (scale.factor = 10000), and identified 2000 hypervariable genes in each sample by “vst” method. We removed batch effects and integrated data using the standard procedures of Seurat v3 (22). We identified the anchors of the data through FindIntegrationAnchors, and integrated the datasets through the IntegrateData function. Then we scaled the data through ScaleData and performd principal component dimensionality reduction on the data through RunPCA (npcs=30). After that, We constructed k-NN graph through FindNeighbors(k.param = 20, reduction = “pca”, dims = 1:30) and performed t-Distributed Stochastic Neighbor Embedding (t-SNE) visualization dimensionality reduction on the data (dims = 1:30). Choosing the resolution as 0.25, we clustered cells by the FindClusters function. Through the FindAllMarkers function, we identified specifically expressed genes in each cell population to assist us in cell type definition (logfc.threshold = 0.1, test.use = “wilcox”), and displayed the top5 highly expressed genes through DoHeatmap. We annotated the cells through the annotation information in literature and known markers. GO enrichment analysis of the differentially expressed genes was implemented using the clusterProfiler (3.12.0) package in R, and analyzed through the enrichGO function (p valueCutoff =0.05,pAdjustMethod = “BH”,qvalueCutoff = 0.2) (23). Cellchat analysis was mainly based on the R package CellChat (version 1.1.3) (24). We used the normal and tumor samples as input sets to construct objects through the CreateCellChat function, and imported the Secreted Signaling database of human ligand receptors in CellChat for analysis. Then, we identified significantly expressed genes by identifyOverExpressedGenes (thresh.fc = 0, thresh. p= 0.05) and identifyOverExpressedInteractions to identify significant interactions.

### Real-time quantitative PCR (qRT-PCR)

Total RNA from tumor samples and normal samples were extracted using RNA Quick Purification Kit (ES Science, Shanghai, China). Complementary DNA (cDNA) synthesis was then carried out using 1 μg of total RNA using the cDNA Reverse Transcription kit (Vazyme, Nanjing, China). QRT-PCR was performed using TB Green™ Premix Ex Taq™ II (RR420A; Takara, China) on a Bio-Rad CFX-96 Real-time PCR system (Bio-Rad, USA), QRT-PCR was run at the following condition: 95°C, 3min; (95°C, 15s; 60°C, 30s;72°C, 30s)×40 cycles, according to the manufacturer’s instructions. All PCR primers for genes are listed in **Supplementary Table 2** and were synthesized by Tsingke Biological Technology (Tsingke, Beijing, China). Relative abundance of mRNA expression was calculated using the 2^−ΔΔCt^ method, and normalized to GAPDH mRNA expression levels.

## Results

### Single-cell transcriptional profiling of ovarian samples and cell-type identification

**Figure 1:**
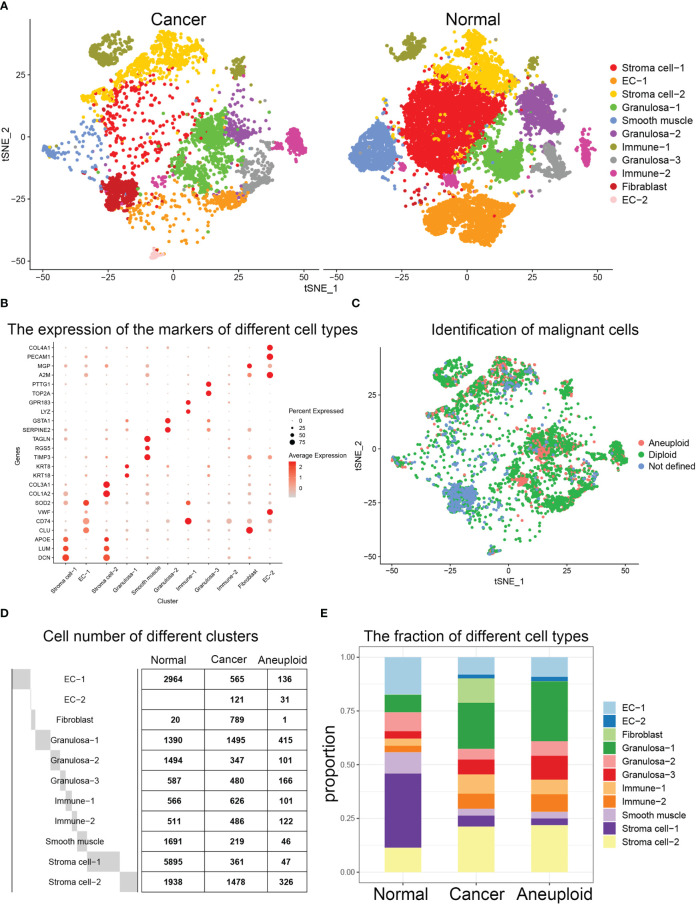
**(A)** Clustering results after integration of the datasets of normal ovary and ovarian cancer samples. t-SNE visualization of the integrated results. **(B)** Dotplot depicting selected marker genes in each cell population. **(C)** Statistics on the number of cells in each group of normal samples and cancer samples. **(D)** Histogram showed the changes in the ratio of cancer samples compared to normal samples. **(E)** tSNE visualization of benign and malignant cells in the tumor dataset.

After integrating the data from healthy ovarian and ovarian cancer samples, the cells clustered into 11 groups, including 6867 tumor cells and 17056 healthy cells (**Figure 1A**, **Supplementary Figure 1**). Dot plots were used to display the marker genes of different clusters, and the characteristics of these genes were used to annotate the cell types (**Figure 1B**). Then, we used the CopyKAT (v1.0.8) to identify the benign and malignant cells in the tumor dataset, in which there were 1492 aneuploid cells (tumor cells) and 4983 cells were defined as diploid cells (normal cells) (**Figure 1C**, **Supplementary Figure 2**). In addition, by calculating the proportions of various cell types, in the ovarian cancer samples, we found a significant decrease in the ratio of Stroma cell−1 and a significant increase in the ratio of Granulosa−1 and fibroblasts (**Figures 1D, E**).

### Differential gene and cell cycle analysis revealed significant functional changes

**Figure 2:**
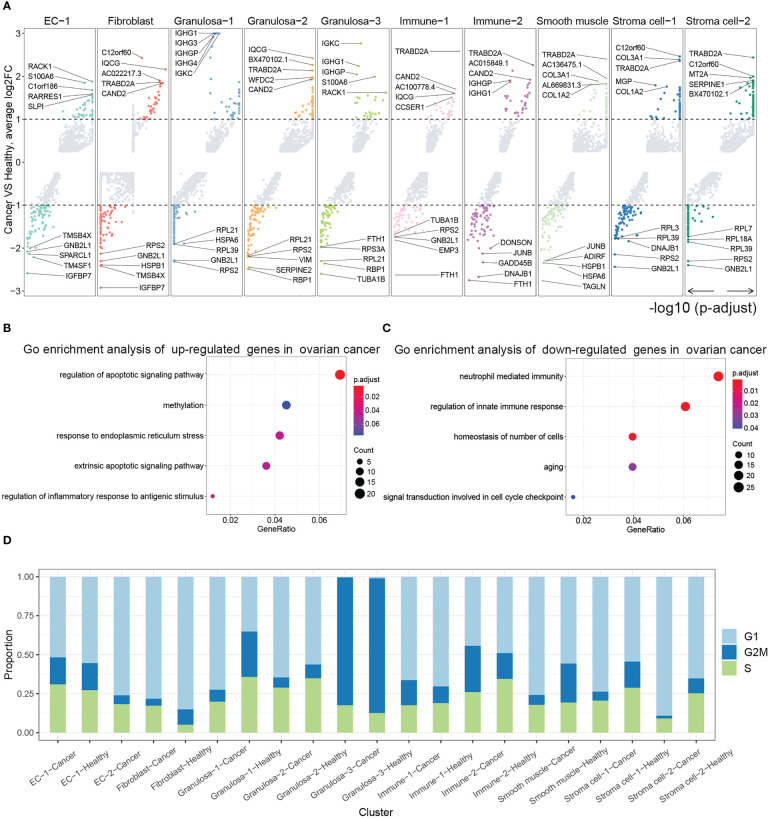
**(A)** Differential analysis between ovarian cancer samples and normal samples. Analysis of the differential genes of each group in ovarian cancer samples compared with normal samples and display of the most significantly up-regulated and down-regulated genes through volcano plots (adjust p value <0.05 and |logFC| ≥1 were set as the cut-off criteria). Go enrichment analysis of **(B)** up-regulated genes and **(C)** down- regulated genes in cancer ovary. **(D)** Histogram showing the proportion of cell cycles for each cell population in normal ovary and ovarian cancer.

We carried out differential gene expression analysis between ovarian cancer and healthy ovarian tissues for each cell population, and found that C1orf60, TRABD2A, CAND2 and other genes were significantly up-regulated genes in multiple clusters (**Figure 2A**). Enrichment analysis of up-regulated and down-regulated genes in ovarian cancer, revealed that up-regulated genes were closely related to apoptosis signaling, inflammatory response, and methylation, while down-regulated genes were closely related to immune system and cell homeostasis (**Figures 2B, C**). Finally, we calculated the proportion of cells in different stages of the cell cycle for each population of ovarian cancer and healthy ovarian cells, and found significant differences among different cell populations. For example, a high proportion of cells in the G2M phase were Granulosa−2 cells (**Figure 2D**).

### Cellchat analysis of ovarian cancer and healthy ovarian tissues revealed different cell interaction patterns

**Figure 3:**
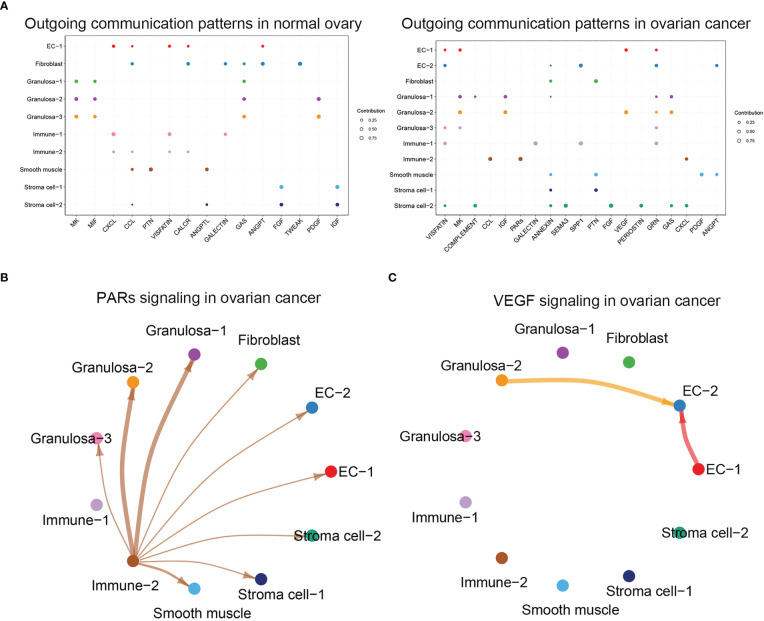
**(A)** Analysis of cellular communication in each cell population in normal ovary and ovarian cancer, dotplot showed the outgoing communication patterns in normal ovary and ovarian cancer. **(B)** Visualization result of PARs signaling in ovarian cancer. **(C)** Visualization result of VEGF signaling in ovarian cancer.

We found significant differences in the communication patterns of different cell groups between healthy ovarian and ovarian cancer tissues using CellChat (**Figure 3A**). Several ovarian cancer cell types generated more signals than healthy ovarian cells, with the cancer cells generating significant levels of PARs and VEGF signals. We found that PARs signaling in ovarian cancer was predominantly generated by Immune-2 and received by various other cell populations (**Figure 3B**). Moreover, VEGF signals were mainly produced by EC-1 and Granulosa cells in ovarian cancer tissues, and EC-2 received the signal, suggesting that the production of EC-2 was closely related to the secretion of VEGF by these two groups of cells (**Figure 3C**).

### Ovarian cancers induced important changes in endothelial cells

**Figure 4:**
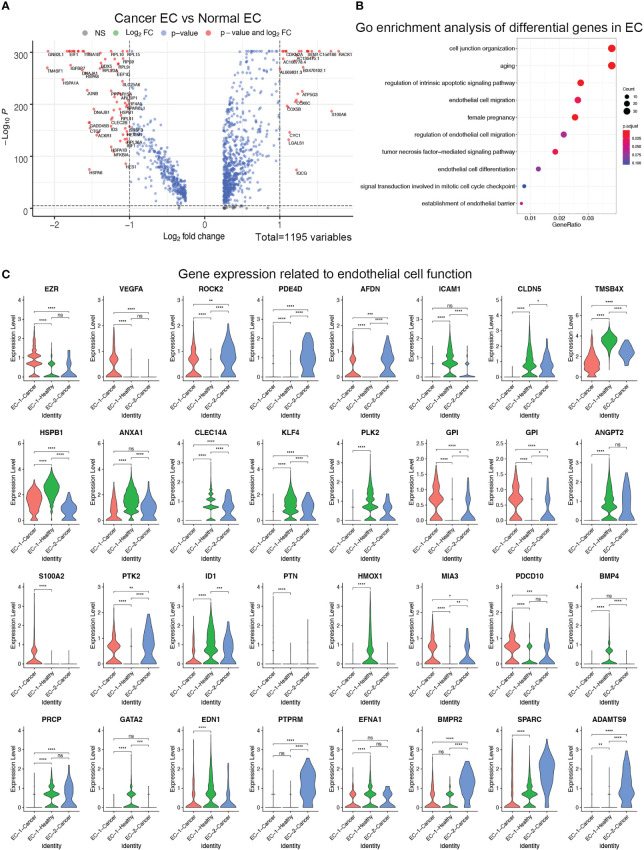
**(A)** Differential analysis of endothelial cells from normal ovary and ovarian cancer. Volcano plot revealed upregulated and downregulated genes in endothelial cells from ovarian cancer versus normal ovary. **(B)** GO enrichment analysis of differential genes between normal endothelial cells and ovarian cancer endothelial cells. **(C)** Violin plots showed the expression of genes related to endothelial cell function in normal endothelial cells and ovarian cancer endothelial cells. ****p > 0.0001; ***p > 0.001; **p > 0.01; *p > 0.05; ns, not significant (P<0.05).

To further explore the difference in endothelial cells between healthy ovarian and ovarian cancer tissues, we performed differential analysis based on two populations of cells, EC-1 and EC-2. A comparison of endothelial cells between healthy ovarian and ovarian cancer tissues revealed that genes such as RACK1, S100A6, and C1orf186 were significantly upregulated, while GMB2L1, TM4SF1, and EIF1 were significantly downregulated in the ovarian cancer samples (**Figure 4A**). GO enrichment analysis on differentially expressed genes showed that the genes were enriched in important pathways related to endothelial cell differentiation, migration, and differentiation (**Figure 4B**). The expression of these genes differed significantly in healthy ovarian and ovarian cancer EC-1 cells, and ovarian cancer-specific EC-2 cells. For example, genes such as VEGFA and EZR were significantly expressed in EC-1-Cancer cells, but genes such as PDE4D and AFDN were not expressed (**Figure 4C**).

### The expression of apoptosis- and immune-related genes was altered in ovarian cancer tissues

**Figure 5:**
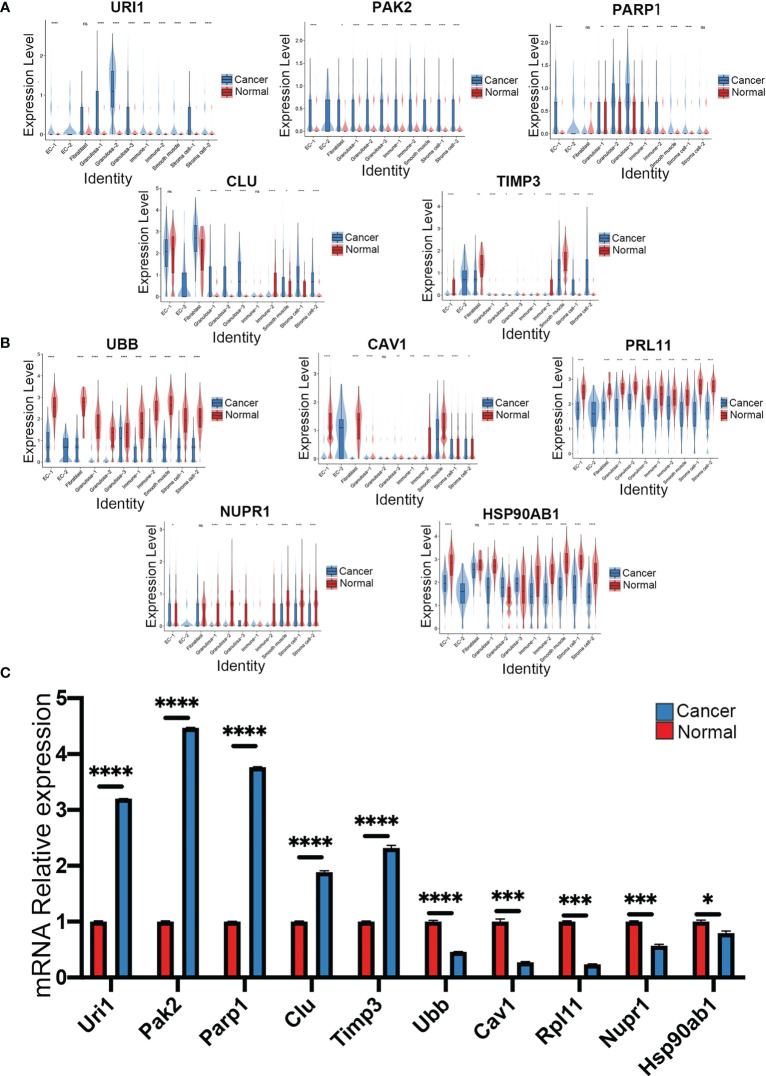
Analysis of apoptosis and immune related pathway genes normal ovary and ovarian cancer, violin plots showed **(A)** apoptosis-related genes and **(B)** immune-related genes in each cluster of the datasets. **(C)** The mRNA expression of ten genes in normal ovary and ovarian cancer was measured by qRT-PCR (****p < 0.0001; ***p < 0.001; **p < 0.01; *p < 0.05, Error bars are ± SEM). ns, not significant (P<0.05).

Based on the results of GO analysis, we further explored the genes related to apoptosis and immunity. We found that apoptosis-related genes URI1, PAK2, PARP1, CLU, and TIMP3 were significantly upregulated in multiple cell populations of cancer cells (**Figure 5A**). However, the immune-related genes UBB, RPL11, CAV1, NUPR1, and Hsp90ab1 were downregulated in multiple cell populations (**Figure 5B**). RT-qPCR analysis revealed that URI1, PAK2, PARP1, CLU, and TIMP3 were significantly upregulated, while UBB, RPL11, CAV1, NUPR1, and Hsp90ab1 were significantly downregulated in the ovarian cancer samples (**Figure 5C**).

## Discussion

The application of ScRNA-seq technology in ovarian cancer research is expected to significantly expand our understanding of the disease. ScRNA-seq in ovarian cancer has led to the identification of different cell types, characterization of tumor heterogeneity, identification of more promising immunotherapeutic targets, and enhancement of our understanding of therapy-induced resistance (25–27). The technology can also be used to identify ovarian cancer stem cells that are important in studying changes in immune pathway-related genes during immunotherapy, to study differences in expression between immunotherapy and immune response, and to provide new insights for the study of tumor exosomes (28, 29). High-grade serous ovarian carcinoma (HGSOC) is the most common histological subtype of ovarian cancer, yet ScRNA-seq has not been extensively used to understand the genetic complexity in high-grade ovarian cancers. ScRNA-seq was used to examine gene expression patterns from single cells of high-grade serous ovarian cancer obtained from a patient. From that study, epithelial and stromal cells were identified as the major subsets based on the RNA expression patterns of 66 evaluable single tumor cells. Findings from the study provided a first glimpse at the application of single-cell gene expression analysis in ovarian cancer to solve the etiology of the disease (30). In another study, single-cell RNA technology revealed the presence of heterogeneity in primary tumor cells among different patients, and differences in the expression profiles between metastatic lesions and primary lesions in different patients (31). Analysis of ascites samples from patients with high-grade ovarian cancer using single cell sequencing identified the JAK/STAT pathway as a therapeutic target in women (32).

Through bioinformatic analysis, we identified several genes associated with ovarian cancer and the signaling pathways associated with the genes. The DNA methylation status has been proven to be a prognostic biomarker for High-grade serous ovarian cancer (33). Our study also demonstrated that up-regulated genes in High-grade serous ovarian cancer were closely associated with methylation levels and were implicated in the inflammatory response. VEGFA is a member of the VEGF family of cytokines that mediates ovarian cancer progression. VEGF is a significant therapeutic target for ovarian cancer since it is highly expressed in the tumor tissues. VEGF inhibitors could have significant therapeutic value in treating ovarian cancer (34). In our research, VEGF signals were significantly enriched in ovarian cancer and VEGFA was significantly expressed in EC-1-Cancer cells. VEGF stimulates endothelial cell proliferation through VEGF receptor 2, which is found on endothelial cells (35).We also found that Ovarian cancers induced important changes in endothelial cells.

QPCR analysis verified the high expression of some genes in human high-grade serous ovarian carcinoma. URI1 may be a ‘non-oncogene’ that supports the oncogenic phenotype of cancer cells that depend on a molecular chaperone system to survive (36). Ovarian cancer cells overexpress or amplify certain R2TP/PFDL subunits, such as URI1, which have been linked to tumour progression (37). Ovarian cancer progression is also mediated by PAK2. The knockdown of PAK2 in ovarian cancer cell lines reduced migration and invasion but had no effect on proliferation or apoptosis, suggesting a possible role for PAK2 in ovarian cancer development (38). The PARP1 inhibitor, rucaparib, has recently been approved by the FDA for the treatment of ovarian cancer (39). Based on findings from this study, PARP1 expression may also contribute to carcinogenesis, in addition to its enzymatic activity (40). Additionally, our findings show the distribution of this gene in ovarian cancer cells, which could be useful for the treatment of ovarian cancer. It is interesting to note that CLU serum levels are elevated in ovarian cancer (41), and that CLU is expressed in malignant tissues of all ovarian cancer patients (42). In our study, we found that CLU was differentially expressed among different cell populations in normal and cancer samples. There is evidence that TIMP3 participates in tumor invasion as well as preferential methylation in ovarian cancer (43), while a similar study showed that TIMP3 mRNA expression was higher in ovarian cancer patients than healthy individuals (44). Findings from the two studies are consistent with our experimental results.

In our study, we found that some genes were down-regulated in cancer tissues compared with normal tissue, suggesting that these genes may play a role in suppressing ovarian cancer development. The expression of UBB is significantly suppressed in certain cancers, including endometrial carcinoma and ovarian cancer (45). This was consistent with our data. UBB is likely to play different roles in different cancer cell types, however, no studies have analyzed the role of UBB. Although RPL11 has not been reported as a cancer suppressor gene in ovarian cancer studies, it is involved in the development of gastric cancer, colorectal cancer, fibroblasts, lymphoma, and esophageal squamous carcinoma. Furthermore, deletion of RPL11 inhibited colon cancer cell death by preventing p53 activation (46, 47). CAV1 plays an oncogenic role in solid tumors, and its expression correlates negatively with tumor invasion. Additionally, CAV1 can be found in the nucleus of ovarian cancer cells (48), suggesting that CAV1 may also inhibit in ovarian cancer. NUPR1 gene plays a variety of roles in benign and malignant tumors. NUPR1 may affect ovarian cancer proliferation and invasion by signaling through the AKT pathway (49). The purpose of our study was to explore the expression of NUPR1 in each cell population in ovarian cancer.

In summary, our ScRNA-Seq data revealed the main cell types and growth processes in the human healthy ovarian tissues. In addition, we showed differences in function, gene expression and cell interaction patterns between ovarian cancer and healthy ovarian tissues for each cell population. These single-cell transcriptome datasets could shed light on major drivers of tumor development and progression. Increased understanding of ovarian cancer at the single-cell level will lead to the development of novel therapies. However, further studies on the functions of the differentially expressed genes in ovarian cancer are required.

## Data availability statement

The original contributions presented in the study are included in the article/**Supplementary Material**. Further inquiries can be directed to the corresponding author.

## Ethics statement

The research protocol was reviewed and approved by the Research Ethics Committee of Sir Run Run Shaw Hospital, School of Medicine, Zhejiang University. The patients/participants provided their written informed consent to participate in this study.

## Author contributions

XZ was mainly responsible for the writing of the manuscript and carried out statistical analyses. SH and CY participated in the designing the study and analysis of data. XS and FS participated in the experiments. JY critically revised the final manuscript and was responsible for the submitted manuscript. All authors contributed to the article and approved the submitted version.
