# Supplementary material for: Comparative analysis between high-grade serous ovarian cancer and healthy ovarian tissues using single-cell RNA sequencing
Source: Front Oncol. 2023 Apr 14;13:1148628. doi: 10.3389/fonc.2023.1148628 (PMC10140397; doi:10.3389/fonc.2023.1148628)
Supplement: Supplementary file 1 [file Table_1.docx]

**Supplementary Table 1** De-identified metadata from 3 ovarian cancer patients and 3 cervical cancer patients

| Patient ID | Age | Race | Diagnosis | Histologic type | Surgical therapy |
| --- | --- | --- | --- | --- | --- |
| PT-1 | 48 | Asian | ovarian cancer | high grade serous carcinoma | cytoreductive surgery for ovarian cancer |
| PT-2 | 50 | Asian | ovarian cancer | high grade serous carcinoma cytoreductive surgery for ovarian cancer | |
| PT-3 | 46 | Asian | ovarian cancer | high grade serous carcinoma cytoreductive surgery for ovarian cancer | |
| PT-4 | 47 | Asian | cervical cancer | normal ovarian tissues | laparoscopic uterine bilateral adnexectomy |
| PT-5 | 45 | Asian | cervical cancer | normal ovarian tissues | laparoscopic uterine bilateral adnexectomy |
| PT-6 | 42 | Asian | cervical cancer | normal ovarian tissues | laparoscopic uterine bilateral adnexectomy |

**Supplementary Table 2** Sequences of primers used for qRT-PCR analysis

| Name | Sequences |  | Product size(bp) |
| --- | --- | --- | --- |
| Uri1 | F :5' TGCTCATAAACCGCATTCCAA3' | R :5' ACCCAGCAATTCTTCCTGTCT3' | 145 |
| Pak2 | F :5' TGAGCACACCATCCATGTTGG3' | R :5' AGGTCTGTAGTAATCGAGCCC3' | 116 |
| Parp1 | F: 5' CGGAGTCTTCGGATAAGCTCT 3' | R: 5' TTTCCATCAAACATGGGCGAC3' | 136 |
| Clu | F: 5' CCAATCAGGGAAGTAAGTACGTC3' | R: 5' CTTGCGCTCTTCGTTTGTTTT3' | 101 |
| Timp3 | F: 5' CATGTGCAGTACATCCATACGG 3' | R:5' CATCATAGACGCGACCTGTCA3' | 100 |
| Ubb | F: 5' GGTCCTGCGTCTGAGAGGT 3' | R:5' GGCCTTCACATTTTCGATGGT 3' | 106 |
| Cav1 | F:5'GCGACCCTAAACACCTCAAC3' | R: 5' ATGCCGTCAAAACTGTGTGTC 3' | 91 |
| Rpl11  Nupr1  Hsp90ab1  GAPDH | F: 5'AAAGGTGCGGGAGTATGAGTT 3'  F: 5'CTCTCATCATGCCTATGCCTACT3'  F: 5'CATCTCCATGATTGGGCAGTT3'    F: 5'CTGGGCTACACTGAGCACC 3' | R:5' TCCAGGCCGTAGATACCAATG 3'  R:5' CCTCCACCTCCTGTAACCAAG3'  R:5' CTTTGACCCGCCTCTCTTCTA3'  R:5' AAGTGGTCGTTGAGGGCAATG 3' | 154  144  233  101 |
