# Supplementary material for: Comparative analysis between high-grade serous ovarian cancer and healthy ovarian tissues using single-cell RNA sequencing
Source: Front Oncol. 2023 Apr 14;13:1148628. doi: 10.3389/fonc.2023.1148628 (PMC10140397; doi:10.3389/fonc.2023.1148628)
Supplement: Supplementary file 2 [file DataSheet_1.pdf]

Supplementary Figure 1

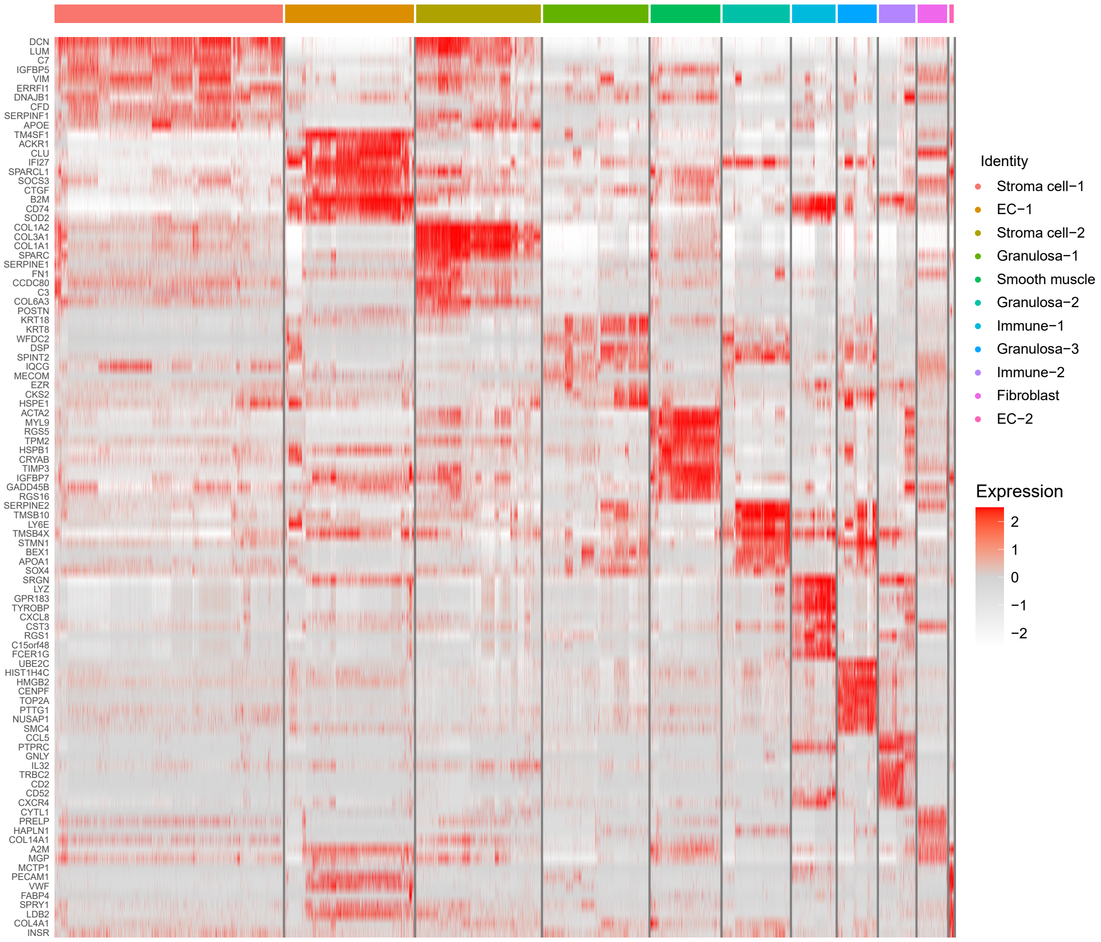

Supplementary Figure 1. Heatmap showing the highly expressed genes specific to each group in the integrated results.

Supplementary Figure 2

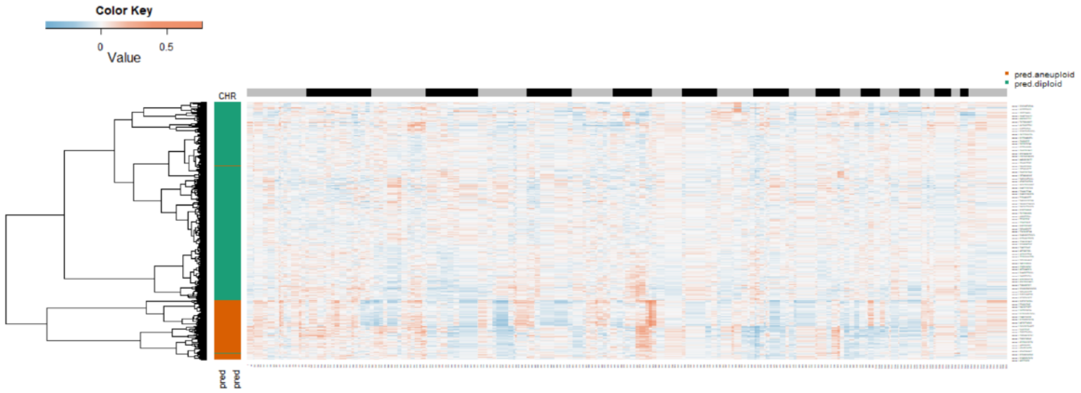

Supplementary Figure 2. Heatmap of CopyKat annotation result of cancer sample.
